# To Match or Not to Match? Reactions to Turning Points in Negotiation

**DOI:** 10.1007/s10726-017-9550-x

**Published:** 2017-11-25

**Authors:** Michele Griessmair, Daniel Druckman

**Affiliations:** 1 Sir Zelman Cowen Centre, Victoria University, Melbourne, Australia; 2 University of Vienna Faculty of Business, Economics and Statistics, University of Vienna, Vienna, Austria; 3 Schar School of Policy and Government, George Mason University, Arlington, VA USA; 4 Department of Modern History, Politics and International Relations, Macquarie University, Sydney, Australia; 5 School of Political Science and International Affairs, University of Queensland, Brisbane, Australia

**Keywords:** Matching, Negotiation, Process frames, Salience of offers, Turning points

## Abstract

This study examines the impacts of process frames and salience of a turning point on negotiators’ responses to a departure during the negotiation process. Results show that individuals negotiating within an integrative-cooperative (as opposed to a distributive-competitive frame) are more likely to interpret the departure as a turning point and match the other’s offer. Similarly, results show that making the departure salient by clearly articulating the intent, content, and function of the turning point offer increases negotiators’ propensity to embrace the mutually beneficial turning point offer. The findings are discussed in light of negotiators’ awareness of events during the negotiation process, their (mis)matching of favorable offers, and relational order theory.

## Introduction

Departures or turning points are clear and self-evident changes from earlier events or patterns (Druckman and Olekalns 2013b). They are salient incidences within the negotiation that potentially alter its course by causing negotiators to change their strategy, the perception of and the relationship with the counterpart, or the negotiation outcome (Druckman et al. 2009; Olekalns and Smith 2005). Departures vary in their level of abruptness and may consist of procedural changes, incorporation of new ideas, or abandoning a give-and-take pattern (Druckman 2001). They typically occur after a period of little or no progress that leaves the parties prone to face an impasse (Druckman 1986) which may precipitate a turning point. In the present study, the turning point consists of a multi-issue offer that signals a willingness to come closer and marks a departure from competitive bargaining with the potential to introduce a new pattern favorable for both parties.

It is important to distinguish between a departure and its consequence. Whereas the departure refers to the event or action per se, the consequence takes into account the direction of the negotiation as a result of the action. The direction can be a progression toward or away from an agreement (Druckman and Olekalns 2013b). Griessmair et al. (2015) have shown that in order to progress toward an agreement, it is crucial that the counterpart respond in kind and matches the turning point offer. Accordingly, in the present study, we explore reactions to a proposed mutually beneficial turning point offer.

**Fig. 1:**
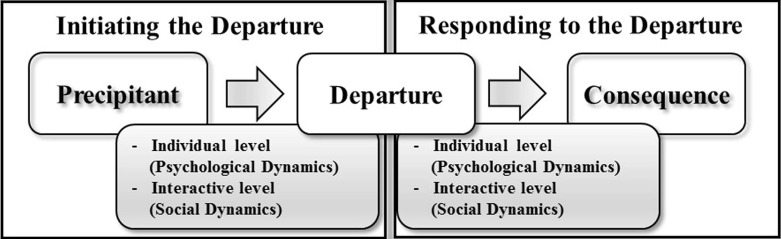
Initiating and concluding the turning point

Summarizing Druckman’s (2001) model, turning points are clear departures from earlier patterns in the negotiation process that are initiated by precipitants and that lead to a consequence (see Fig. 1). However, turning points are neither initiated nor concluded by a single negotiator (Griessmair et al. 2015): the parties are highly dependent on each other for achieving their goals (Lewicki et al. 1999). When a negotiator introduces a departure, the other party may choose to incorporate or ignore the proposed changes. Prior research shows that negotiators’ reactions depend on the psychological and social dynamics of the negotiation (Druckman 2004; Druckman and Olekalns 2013b) (see Fig. 1). Thus, a central question is why some negotiators embrace the departure and direct the negotiation toward an agreement whereas others fail to seize the opportunity.

Two studies have addressed the ways that substantive events or actions produce change in the negotiation process. Investigating reactions to a crisis, Druckman and Olekalns (2013a) show that negotiators with unattractive alternatives are more likely to reframe the issues whereas negotiations with high transaction costs increase the preference for continuing the talks. Shared identity, on the other hand, creates a robust relationship that is not affected by crises. This study highlights the importance of the context in which a crisis occurs. Similarly, focusing on initiating the turning point (left-hand side of Fig. 1), Druckman et al. (2009) ask the question why certain precipitants trigger departures while others do not. They find that crises result in more movement when the social climate is characterized by high trust and low power dependency.

Both studies show that positive change is contingent on psychological and social dynamics affecting negotiators’ interpretation of and reaction to substantive events. As a result, these investigators conclude that in order to better understand turning points, social and psychological processes embedded in the negotiation process need to be considered. Extending this research, the present study addresses the right-hand side of Fig. 1 by investigating the extent to which the process frame and the salience of the departure promote or hinder obtaining a mutually beneficial departure.

## Process Frames

The importance of negotiators being “in sync” or “out of sync” with each other has been demonstrated in various domains including strategies and tactics, goals, linguistic styles, and nonverbal behavior (Maddux et al. 2008; Olekalns and Smith 2000; Taylor and Thomas 2008; Weingart et al. 1999). Although synchronized responding to the turning point offer can propel negotiators towards an agreement (Druckman 2004; Griessmair et al. 2015), studies have also shown that negotiators may engage in mismatching. Negotiators tend to make high demands and low concessions when the counterpart’s behavior is interpreted as conciliatory—the larger the counterpart’s concessions, the smaller the negotiator’s own concessions (Bateman 1980; de Dreu et al. 1994; Druckman and Bonoma 1976; Druckman et al. 1972; Pruitt and Syna 1985). Based on aspiration theory (Siegel and Fouraker 1960), the rationale behind mismatching is that conciliatory negotiators are likely to appear soft, hence, the other party can place high demands without risking an impasse (Van Kleef et al. 2004a).

The choice to match or mismatch, however, may depend on the context of the negotiation (Allen et al. 1990; Carnevale and Pruitt 1992; Kleef et al. 2010). Notably, (mis)matching is not necessarily rooted in the counterpart’s actual behavior (e.g., concessions) but rather in the negotiator’s interpretation of the counterpart’s behavior (de Dreu et al. 1994; Van Kleef et al. 2004a). Allen et al. (1990) showed that a negotiator’s willingness to reciprocate a conciliatory offer depended on whether the concession is perceived as a sign of cooperation. If this is the case, the “norm of reciprocity is more likely to surface” (Allen et al. 1990: 92) and negotiators are more likely to respond in kind. Following this reasoning, we suggest that the likelihood that bargainers will match a turning point offer depends on whether it is proposed within a distributive-competitive or integrative-cooperative process frame.

A distributive-competitive negotiation frame is characterized by an emphasis on one’s own goals regardless of the counterpart’s needs (Putnam 1990) and increases the prominence of a negotiator’s relative gains approach to bargaining (Griessmair and Seferagic 2014). This competitive focus also increases negotiators’ fear that the counterpart may possibly exploit them. On a communicative level, the interaction focuses on arguments to substantiate one’s own position, direct negative reactions, threats, reference to power, and the use of pressure tactics (Weingart et al. 2004). When the departure is proposed within such a frame, the counterpart is less likely to interpret it as a genuine attempt to match concessions but rather allege selfish motivation and strategic behavior (cf., Kleef et al. 2010). As a result, s/he should also be more likely to continue with the previous offer pattern and not adapt his/her behavior to the proposed departure, despite the fact that the turning point offer would result in positive consequences for both.

An integrative-cooperative negotiation frame is characterized by a joint problem solving orientation that focuses on the identification of shared objectives and similarities (Putnam 1990). The emphasis is on the underlying interests of both parties, highlighting mutuality, and engaging in conciliation and constructive exploration (Weingart et al. 2004). Moreover, the exchange of information focuses on priorities rather than positions (Weingart et al. 2004), which has a positive impact on the negotiators’ relationship by generating trust (Liu et al. 2010). This integrative-cooperative frame steers a negotiator’s attention toward working together for a mutually beneficial outcome (Gelfand et al. 2006; Griessmair and Seferagic 2014). Thus, a counterpart’s new offer is more likely to be assessed in light of possible mutual gains and areas of agreement. Furthermore, an integrative-cooperative frame also increases negotiators’ perceived fairness (Griessmair and Seferagic 2014) which, in turn, increases the likelihood that negotiators exhibit reciprocal behavior and engage in matching rather than mismatching (Pruitt and Syna 1985; Smith et al. 1982). When the turning point offer is proposed within an integrative-cooperative as opposed to a distributive-competitive frame, it is more likely that the suggested departure is interpreted as a genuine attempt to turn the negotiation around and the counterpart responds in kind (cf., Allen et al. 1990).

Further support for the idea that the process frame influences how individuals interpret and react to departures is provided by relational order theory (ROT) (Donohue 1998; Donohue and Hoobler 2002; Donohue and Roberto 1993). ROT posits that negotiators continuously create and tacitly negotiate relational orientations with implications for the substantive negotiation process (Donohue 1998). Depending on the degree of conveyed affiliation and interdependence, they establish a relational context of ‘moving toward/with’ or ‘moving against/away’ (Donohue and Roberto 1993). By its joint problem solving orientation and focus on shared objectives, an integrative-cooperative process frame signals affiliation, thus establishing a moving towards/with relational context. Conversely, a distributive-competitive emphasis on one’s own goals, regardless of the counterpart’s needs, is more likely to create a moving against/away relational context.

Most importantly, according to ROT, the relational context constitutes the groundwork for substantive negotiation behavior. Moving toward or with facilitates crafting integrative solutions whereas moving against or away leads negotiators to “experience significantly more difficulty in reaching satisfying agreements” (Donohue 1998: 80). Thus, ROT suggests that the relational context created by an integrative-cooperative (as opposed to a distributive-competitive) process frame creates a conducive foundation for departures in the negotiation.

Thus, we hypothesize that proposing the turning point offer within an integrative-cooperative frame increases the likelihood that the turning point offer is seen as a genuine attempt to turn the negotiation around. This then increases the likelihood that the counterpart will respond in kind to the mutually beneficial departure. Two hypotheses are suggested.

**H1a**: Proposing the turning point offer within an integrative-cooperative (versus a distributive-competitive) frame increases the likelihood that the turning point offer is interpreted as such by the counterpart.
**H1b**: Proposing the turning point offer within an integrative-cooperative (versus a distributive-competitive) frame increases the likelihood that the counterpart embraces the turning point offer and adapts his/her offer pattern to the proposed departure.

## Salience

A defining feature of turning points is that they are clear and self-evident departures from previous events and patterns (Druckman and Olekalns 2013b), that is, the involved parties have to be aware that they have occurred. Accordingly, Druckman points out that the “key element is that change, often initiated by one of the parties, is manifest in the interaction process” (2004: 191). This requires that the negotiators keep track of the negotiation process in order to detect deviations from previous negotiation patterns and stay alert to concessions that require adjustments. Also, awareness of the counterpart’s strategy shift is necessary to adjust to the shift (Druckman et al. 1983). A number of studies suggest that this might not necessarily be the case during the actual negotiation process (e.g., Keysar et al. 1995; Boven et al. 2003; Vorauer and Claude 1998). Hence, a clear departure such as proposing a trade-off that increases the value for both parties after a period of competitive bargaining may go unnoticed by the counterpart and, as a result, not lead to a beneficial consequence.

Negotiators have limited information about their counterpart’s needs and interests but commonly “know what they want and assume the other party wants the opposite (the fixed-pie bias)” (Adair and Brett 2005: 35). An accurate understanding of the counterpart’s priorities and needs is crucial in mixed-motive negotiations. Being aware of the other’s preference structures is not only relevant for proposing trade-offs and concessions but also for recognizing them as such. Kersten et al. (2000) note that an offer intended by one party as a concession may be perceived by the other party as a reverse concession (see, also, Wachowicz and Wu 2010). Although this can be ameliorated by effective information exchange (e.g., Adair et al. 2004; Liu et al. 2010; Thompson 1991), a number of studies show that negotiators do not communicate as effectively as they think they do (e.g., Keysar et al. 1995; Boven et al. 2003; Vorauer and Claude 1998). Rather than addressing the informational needs of their counterpart when making an offer, negotiators tend to overestimate the transparency of their goals and behave as if the counterpart has access to their privileged information. For instance, Boven et al. (2003) show that business students experienced in negotiation overestimated their success in communicating information about their preferences and Vorauer and Claude (1998) find that negotiators have the tendency to overestimate the extent to which their counterpart can discern their joint problem solving approach. Similarly, Bazerman and colleagues conducted a series of studies (e.g., Bazerman and Carroll 1987; Keysar et al. 1995; Samuelson and Bazerman 1985) showing that negotiators failed to include information about their counterpart’s access to information in their assessment of his or her decision.

Both negotiators’ limited information about the counterpart and ineffective information exchange hinders negotiators from detecting deviations from established patterns. As a result, a beneficial offer with the potential to redirect the negotiation after a period of slow progress may go unnoticed by the counterpart and not lead to the intended consequence. Previous research has shown that a higher degree of communication clarity during the negotiation process enables the involved parties to better understand each other’s interests, preferences, and priorities which, in turn, aids negotiators to develop more options and find mutually beneficial solutions (Liu et al. 2010). Accordingly, a higher degree of clarity results in superior negotiation outcomes and more cooperative responses (e.g., Adair et al. 2004; Lindskold et al. 1986; Liu et al. 2010; Thompson 1991). Similarly, Allen et al. (1990) highlight the critical function of communication in bargaining. In particular, they emphasize conveying the cooperative intent of an accommodating and conciliatory offer in order to induce reciprocal behavior in the counterpart. Thus, we expect that making the departure salient by clearly articulating the intent, content, and function of the offer increases the likelihood that it will be interpreted as intended and reciprocated by the counterpart. Two hypotheses are suggested.

**H2a**: Making the departure salient increases the likelihood that the turning point offer is interpreted as intended by the counterpart.
**H2b**: Making the departure salient increases the likelihood that the counterpart adapts his/her offer pattern to the proposed departure.

As discussed in the previous section, an integrative-cooperative frame increases negotiators’ perceived fairness of the process and the counterpart, steers their attention toward the cooperative potential of the negotiation, and fosters the motivation to work together to find a mutually beneficial outcome. As a consequence, individuals negotiating within an integrative-cooperative frame should be more receptive to a message highlighting mutual benefits. Thus, we expect that the effects of salience are stronger in the integrative-cooperative frame as summarized by the following hypothesis.

**H3**: The effects on cooperation of making the departure salient are stronger within an integrative-cooperative negotiation frame.

## Method

This section is divided into five parts: participants and design, tasks, procedures, independent variables, and dependent variables.

### Participants and Experimental Design

A total of 112 graduate students participated in the study, 5 of which were eliminated because they ended the negotiation prematurely, resulting in a sample size of 107. All participants were recruited from negotiation courses in which they conducted simulated negotiations with payoff tables in order to illustrate concepts such as trade-offs, joint gains, and logrolling. For participation they received additional grade points and participated in a lottery. Participants were told that they would be negotiating online against someone from another university. They were assigned the role of seller, received identical role instructions, and received the same offers from their alleged counterpart.

The $2 \times 2$ design consisted of four treatments with two variables (Table 1): the process frame (distributive-competitive vs. integrative-cooperative) and the salience of the turning point offer (the turning point offer was or was not made explicit).

**Table 1:** Overview of the treatments

	Process frame	
	Distributive competitive	Integrative cooperative
Salience		
No TP-Message	n $=$ 26	n $=$ 27
TP-Message	n $=$ 28	n $=$ 26

With each offer, the alleged counterpart sent a short message that was used to manipulate the process frame and salience. The participants could also choose between pre-formulated messages that were identical in all treatments. After the negotiation, participants completed a post-questionnaire that included general questions about the negotiation, the counterpart and the outcome as well as questions specifically addressing the turning point.

In addition to receiving course credits, participants took part in a lottery in order to increase their involvement. They were told that each payoff point would be converted into a lottery ticket and the more lottery tickets they obtained the higher the chance of winning either €50 or an iPod Shuffle of approximately equivalent value. In total, four prizes were distributed. However, only those participants that reached an agreement would participate in the lottery. This highlights the mixed-motive nature of the negotiation: the participants have an incentive to reach an agreement as well as to achieve as many payoff points as possible.

### Negotiation Task

The negotiation task consisted of the negotiation of tablet PCs with three issues: price, license contract duration, and online storage. It captured the main characteristics of a real life negotiation such as receiving no information about the counterpart’s utilities and multiple issues differing in utility.

The participants’ role instructions also included a payoff matrix indicating the relative importance of the three issues. The payoff matrix was adapted from Van Kleef et al. (2004a, b) and all three issues had 9 payoff levels with level 1 yielding 0 payoff-points and level 9 the maximum payoff-points for the respective issue (price: 400; license contract duration: 120; online storage: 240). The role instructions included an aspiration level and a bottom line for each issue. Furthermore, participants were told that they should try to reach an agreement but at the same time achieve as many payoff points as possible. They received no information about the alleged counterpart’s payoffs.

The concessions of the alleged counterpart in the initial steps followed the pattern employed by Van Kleef et al. (2004a, b) and de Dreu and Lange (1995) consisting of concessions of one step in the participant’s payoff matrix on a single issue. In the 4th counteroffer, the alleged counterpart introduced the turning point offer. As described above, turning points are clear departures from earlier patterns in the negotiation such as abandoning a give-and-take pattern or introducing new multi-issue packages. Accordingly, our turning point offer abandoned the incremental single-issue concession pattern and introduced a multi-issue offer suggesting potential trade-offs and a large concession. Specifically, the turning point offer consisted of a multi-issue offer with a large concession on a high priority issue for the participants (2 steps on price), a minor concession on a medium priority issue (1 step on online storage), and claiming value on a low priority issue (1 step on license contract duration).

Thus, the turning point offer consisted of a substantial concession that signals willingness to accommodate in order to reach an agreement and also introduced potential trade-offs. Whereas in the previous offers by the alleged counterpart only concessions on a single issue were made, the turning point offer involved all three issues including trade-offs, potentially providing information about the preferences of the alleged counterpart. The structure of the multi-issue offer—conceding on a high priority issue for the participant and claiming value on a low priority issue—signals that the alleged counterpart has a payoff structure with different priorities and consequently the potential to make trade-offs, the possibility of increasing joint gains, and exhibits an integrative feature of negotiation. Furthermore, the large concession on the other’s high priority issue signaled a willingness to come closer, which may be considered as a hallmark of turning point offers.

Following Druckman’s (2001) framework, the turning point offer has to be incorporated in order to result in positive consequences. That is, the counterpart has to confirm the turning point offer by changing the structure of the counteroffer and adapting his/her concession behavior accordingly. Notably, this does not necessarily imply larger overall concessions but altering the structure of the offer: Individuals confirming the turning point offer as a pattern for the remainder of the negotiation should make larger concessions on license contract duration—the issue the alleged counterpart signaled to be of high priority by claiming value in the turning point offer—and make smaller concessions on issues the alleged counterpart accommodated substantially.

In the remaining two steps, the alleged counterpart does not concede on license contract duration, further suggesting that this issue is of high priority for him or her. In the step after the turning point (5th step), the alleged counterpart concedes on online storage (medium priority issue for participants) and in the final step the alleged counterpart concedes on price (high priority issue for participants). Overall, the turning point offer is the largest concession, in terms of payoffs, made by the alleged counterpart.

### Procedures

Upon arrival at the laboratory, subjects were seated in individual cubicles equipped with a computer. In each session, the maximum number of allowed participants was 15. They were informed that the negotiation will take place via instant messaging and that they would negotiate with students from another university in a different country (Australia or USA). To enhance the awareness of a ‘real’ counterpart, the instructor referred to the participants of the other university several times during the instructions.

After the general instructions, participants received two handouts. The first contained information about the general setting and the computer program and the second contained the role instructions. The participants first read each handout individually and subsequently received a short briefing including the possibility to ask questions. Each session, including instructions and post-questionnaire, lasted for approximately one hour.

**Fig. 2:**
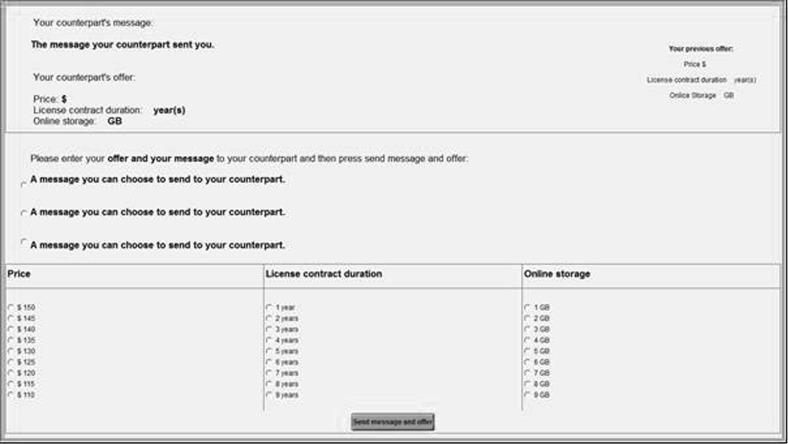
Offer screen

All participants were assigned the role of seller and made the first offer with the counterpart being a computer program implemented in z-Tree (Fischbacher 2007). In each step, participants had to make an offer on the three issues and could choose between three pre-formulated messages to send along with their offer (Fig. 2). On the top of the screen, their own and the alleged counterpart’s previous offer and message was displayed. The three messages the participants could choose from consisted of a neutral (“Thank you for your offer. Here is my return offer.”), an integrative (e.g., “I believe we can find trade-offs to increase the gain for both of us.”), and a distributive (e.g., “I will only concede if I get something in return.”) statement. Participants were told that they should choose the message they regard as the most appropriate reply. The sequence with which the pre-formulated messages were displayed on the screen was randomized. After having sent the offer, a waiting screen with the alleged counterpart’s previous offer was displayed. In order not to arouse suspicion, the waiting time for the counteroffer was determined by a Poisson distribution with an expected value of two minutes.

**Fig. 3:**
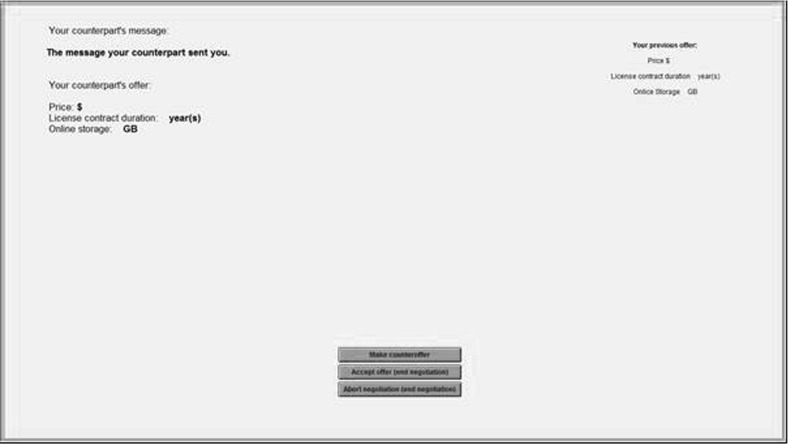
Information screen after alleged counterpart’s counteroffer

The information screen (Fig. 3) of the alleged counterpart’s offer displayed the respective counteroffer and message as well as the participant’s previous offer. Furthermore, the participants could decide whether to make a counteroffer, abort the negotiation, or accept the offer. If they chose to make a counteroffer, they proceeded to the offer screen (Fig. 2).

The participants were told the negotiation will end when either an agreement was reached (accept) or one party decided that an agreement is not possible (abort). If the participants did not accept or abort the negotiation earlier, the negotiation ended with an acceptance message by the alleged counterpart in the 7th round. The number of steps of the negotiation is based on the evidence that after the 6th step the participants start suspecting that they are not negotiating against a real counterpart (de Dreu and Lange 1995). After the negotiation ended, the participants proceeded to answer post-negotiation questions.

### Independent Variables

As described above, we hypothesized that the process frame and the salience of the turning point offer affects whether the departure is interpreted as such and confirmed by the counterpart. In order to investigate the proposed hypotheses, we kept the substantive aspect of the negotiation constant and manipulated the process frame and the salience via the messages the alleged counterpart sent. All participants received the same information about the negotiation task and the counterpart as well as identical offers and concessions, including the turning point offer. Thus, the departure from previous negotiation patterns had the same value for all participants.

#### Process Frames

Since we needed to keep the substantive aspect of the negotiation constant in order to guarantee identical turning point offers, we manipulated the process frame via the communicative behavior of the alleged counterpart. In the integrative-cooperative treatment the offers and concessions were accompanied by messages reflecting integrative strategies. In the distributive-competitive treatment the messages sent by the alleged counterpart reflected distributive strategies. For instance, while the economic value of the 3rd counteroffer was the same for all participants, in the integrative-cooperative frame the accompanying message highlighted flexibility (“I am willing to be flexible on some issues if you can do the same.”) and in the distributive-competitive treatment the message phrased the same concession as insisting on a position (“If you insist on your offers I have to do the same.”).

The messages were constructed based on studies using or explaining quantitative coding of integrative and distributive strategies (e.g., Adair 2003; Olekalns and Smith 2000; Weingart et al. 2007). Examples given in these studies were used whenever possible. Furthermore, the integrative and distributive statements were mirrored conveying comparable substantive content (e.g., “If you could agree on the following offer, an agreement is in sight.” vs. “If you don’t agree to the following offer, we won’t reach an agreement.”).^1^

#### Salience

The salience of the turning point was manipulated by sending a message that made the departure verbally explicit. In two treatments the participants received a message resembling the previous messages (In the integrative-cooperative condition: “I can come closer to your offer. We both are interested in the deal so let’s create something that is good for both of us.” In the distributive-competitive condition: “I can make you the following offer but this is my bottom line. I am only interested in a good deal and I require a good offer.”). Thus, the communication followed the previous pattern and no direct indication for a departure was given.

Conversely, in the other two conditions the participants received the following message with the turning point offer: “I just suggested a trade-off and made a large concession on an issue that seems very important to you. Take a look, I think this offer is good for both of us and might turn the negotiation around.” The message made the potential departure salient by explicitly highlighting that the offer includes (1) a potential trade-off that is (2) beneficial for both parties with (3) a large concession on a priority issue of the participants and (4) a direct referral to a departure with the potential to turn the negotiation around. Thus, in addition to receiving an offer representing a turning point at the payoff-level, the structure and the intention of the turning point offer was made salient at the communicative level.

### Dependent Variables

After the negotiation, participants completed a post-questionnaire. It contained questions about the negotiation, the counterpart, and the outcome, as well as questions specifically addressing turning points. Thus, we assess perceptions of the departure capturing whether the development of the negotiation process, the (alleged) counterpart’s behavior, and the proposed offers were interpreted by the participants as turning points. The questions referring to the turning point were mixed with the general negotiation outcome measures in order not to reveal the intent of the study. The post-questionnaire also contained items about experienced competitiveness used as manipulation checks.

#### Offer’s Payoff

In each step, participants had to make an offer on the three issues. Each issue had 9 payoff levels with level 1 yielding 0 payoff-points and level 9 the maximum payoff-points for the respective issue (price: 400; license contract duration: 120; online storage: 240).

#### Development of the Negotiation Process

Participants were presented with five statements describing the development of the negotiation process and were asked to choose the message that best characterized the negotiation they just conducted. One of the statements, number 2, represented a departure from zero-sum bargaining and an attempt to come closer introduced by the counterpart by making a substantial concession and suggesting a trade-off. The five statements are: (1) Our positions were very similar already at the start of the negotiation and we only had to make minor modifications in the offers; (2) At the beginning our offers were far apart, but then my counterpart made the first step by making a substantial concession and suggesting a trade-off (departure); (3) At the beginning our offers were far apart, but then I made the first step by making a substantial concession and suggesting a trade-off; (4) At the beginning our offers were far apart and progress towards a middle ground was slow, and (5) We made little to no progress until the end of the negotiation.

#### Counterpart’s Turning Point Behavior

Participants were asked to evaluate their (alleged) counterpart’s behavior on a scale from 1= totally disagree to 7=totally agree. Four out of the ten questions addressed the counterpart’s behavior associated with turning points: (1) My counterpart came closer to my position by making concessions and suggesting trade-offs; (2) My counterpart’s behavior directed the negotiation towards positive grounds; (3) My counterpart understood my priorities (what my most important issues are) and acted accordingly, and (4) My counterpart was willing to compromise on his/her initial position. The four items were averaged into a single measure. The measure exhibits a satisfactory Cronbach’s alpha ($\upalpha = 0.83$).

#### Offers and Counteroffers

Participants were asked to evaluate the (alleged) counterpart’s offers and counteroffers on a scale from 1= totally disagree to 7=totally agree. Four items directly addressed characteristics of turning point offers: (1) My counterpart was willing to compromise on my most important issue; (2) My counterpart made higher offers at the beginning but then made appropriate concessions; (3) At some point in the negotiation, my counterpart made an offer that turned the negotiation around, and (4) The offers my counterpart made did not show any willingness to come closer to my position (reverse scaled). The four items were averaged into a single measure. The measure exhibits a satisfactory Cronbach’s alpha ($\upalpha = 0.66$).

#### Communication Influence

Finally, we specifically assess whether the participants perceived that the messages influenced their behavior and judgements. Four of the 12 items addressed turning points: Because of the messages my counterpart sent me ... (1) I understood when my counterpart made a concession; (2) I knew when my counterpart was trying to direct the negotiation towards positive grounds; (3) I understood when my counterpart made a concession, and (4) I knew when my counterpart was trying to direct the negotiation towards positive grounds. The four items were measured on a scale from 1= totally disagree to 7  =  totally agree and were averaged into a single measure. The measure exhibits a satisfactory Cronbach’s alpha ($\upalpha = 0.85$).

## Results

Following the checks on the manipulations, we evaluate the hypotheses on process frame and the salience of the turning point offer.

### Manipulation Checks

In order to test whether the manipulation was successful—that is, whether the participants receiving integrative phrased messages perceive the negotiation as more cooperative-integrative than participants confronted with distributive worded messages—we perform a manipulation check based on items in the post-questionnaire. The six items were used to measure the degree of integrative-cooperative as opposed to competitive-distributive negotiation process frame. The items were measured on a scale from 1= totally disagree to 7=totally agree and averaged into a single measure. The measure exhibits a satisfactory Cronbach’s alpha ($\upalpha$ = 0.70). Results of a *t*-test show that participants in the integrative-cooperative treatment (mean = 5.24, SD = 0.84) perceived the negotiation as more integrative and cooperative than individuals in the distributive-competitive treatment (mean = 3.98, SD = 0.83, $p<$ 0.01) indicating that the manipulation via the messages was successful. The salience manipulation employs an explicit statement directly affecting the dependent variables of interest. Thus, the manipulation is evidenced by the outcomes reported in the next section.

### Negotiator’s Interpretation of the Departure

In order to investigate the effects of our manipulations on the negotiators’ assessment and interpretation of turning point characteristics, we performed a $2\times 2$ ANOVA with process frame (integrative-cooperative vs. distributive-competitive) and salience as factors. We also calculated Cohen’s *d* as a measure of effect size. The results can be found in Table 2.

**Table 2:** Negotiators’ interpretation of the turning point characteristics$^{\mathrm{i,ii,iii}}$

	Distributive-competitive frame		Integrative-cooperative frame		Cohen’s *d*frame	Cohen’s *d*TP-Message
	No TP Message	TP Message	No TP Message	TP Message		
Negotiation development	23.1 %$_{\mathrm{a}}$	28.6 %$_{\mathrm{a}}$	55.6 %$_{\mathrm{b}}$	65.4 %$_{\mathrm{b}}$		
Counterpart’s behavior	3.59$_{\mathrm{a}}$	4.17$_{\mathrm{a}}$	5.06$_{\mathrm{b}}$	5.66$_{\mathrm{b}}$	1.24	0.40
	(1.34)	(1.29)	(1.08)	(0.82)		
Communication influence	3.96$_{\mathrm{a}}$	4.65$_{\mathrm{a}}$	5.41$_{\mathrm{b}}$	5.84$_{\mathrm{b}}$	1.34	0.46
	(1.05)	(1.11)	(0.77)	(0.75)		
Offers and counteroffers	3.98$_{\mathrm{a}}$	4.40$_{\mathrm{a}}$	5.30$_{\mathrm{b}}$	5.68$_{\mathrm{b}}$	1.17	0.29
	(1.43)	(1.25)	(0.81)	(0.72)		

$^{\mathrm{i}}$ Standard deviations in parentheses

$^{\mathrm{ii}}$ Cells sharing the same subscript are not significantly different from each other at the 95 % level

$^{\mathrm{iii}}$ The percentages for the variable negotiation development indicate the proportion of participants that have chosen the turning point scenario in the respective treatment

#### Development of the Negotiation Process

Table 2 shows the percentages of participants that chose the turning point offer as the one best characterizing the negotiation they just conducted. Despite being confronted with the same development of utilities in terms of offers and concessions, a significantly higher number of individuals negotiating within an integrative-cooperative process frame chose the turning point offer as the one best characterizing the negotiation they just conducted ($\chi ^{2} = 12.95; p < 0.01$). Although more negotiators chose the turning point scenario when the departure was made salient with a message, the difference is not significant ($\chi ^{2} = 0.49; p > 0.1$).

#### Counterpart’s Turning Point Behavior

Results show significant effects for the process frame ($F= 43.84; p< 0.01$) and salience ($F = 7.04; p< 0.01$) manipulations. The interaction effect was not significant ($F = 0.01; p> 0.1$). When negotiating within a cooperative-integrative frame and when the turning point offer is made salient, negotiators are more likely to perceive the counterpart as exhibiting behavior characteristic of turning points such as moving the negotiation in a positive direction and understanding the negotiators’ priorities and acting accordingly.

#### Offers and Counteroffers

Results show significant effects for the process frame ($F = 37.65; p< 0.01$) and a borderline effect for salience ($F = 3.64; p < 0.1$). The interaction effect between process frame and salience is not significant ($F = 0.01; p > 0.1$). Thus, individuals negotiating within an integrative-cooperative process frame—and to some extent when the turning point offer is made salient—are more likely to interpret the (alleged) counterpart’s offers as being characteristic of a turning point.

#### Communication Influence

Results show a significant effect for the process frame ($F = 52.98; p< 0.01$) and salience ($F = 9.59; p < 0.01$). The interaction effect was not significant ($F = 0.52; p >0.1$). Thus, both integrative-cooperative communication and making the departure salient increases a negotiator’s understanding of the counterpart’s intention when attempting to direct the negotiation in a more positive direction.

### Responding to Turning Point Offers

**Fig. 4:**
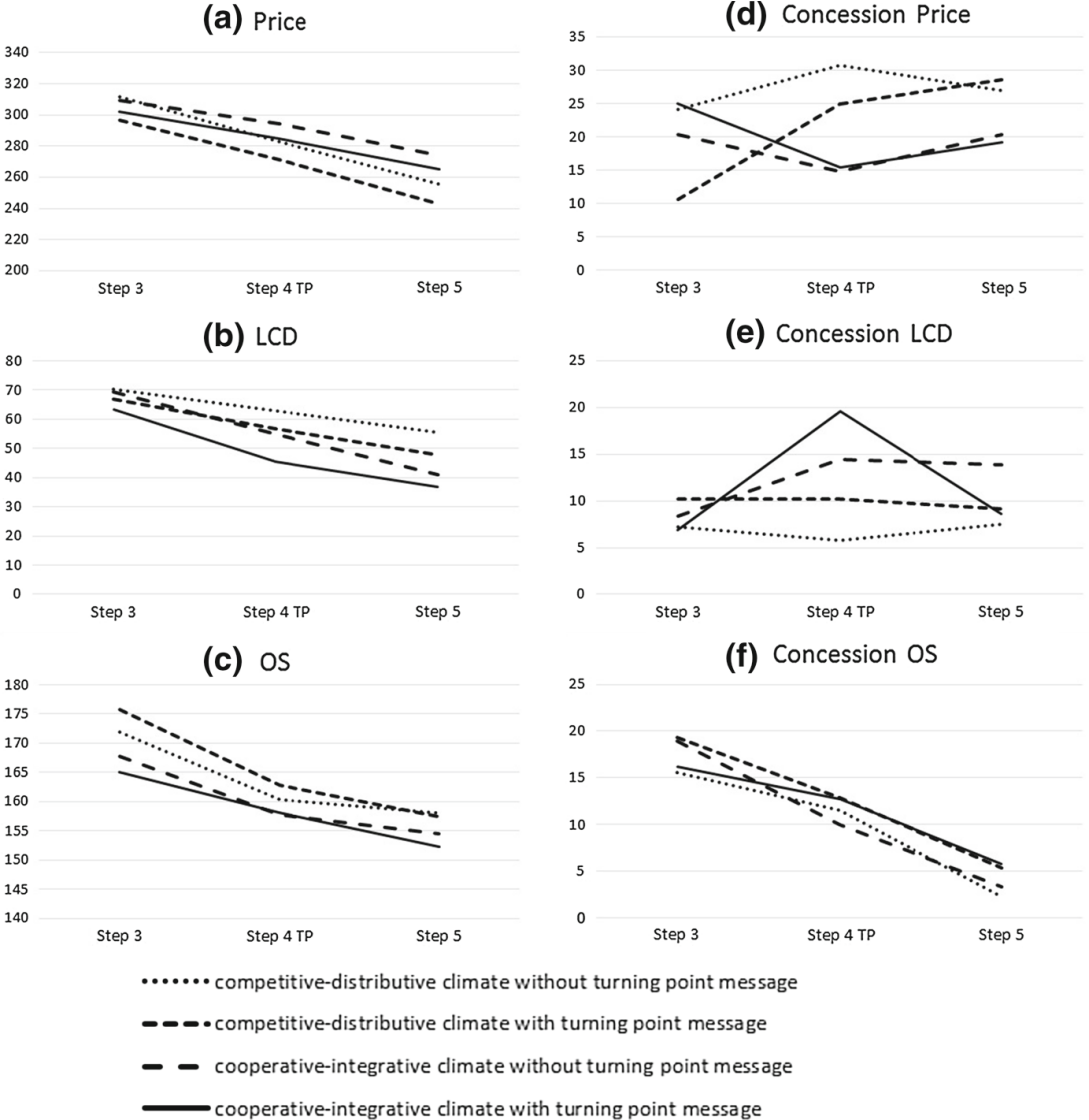
Offers and concessions at the turning point

In order to investigate the effects of our manipulations on the negotiator’s counteroffer, we calculated a 2*2 ANOVA with process frame and salience as factors. All reported analyses were bootstrapped with 1000 repetitions. Fig. 4a–c show the payoff development and Fig. 4d–f show the development of the concessions for all three issues from step 3 to step 5, with step 4 being the participants’ counteroffer to the turning point offer. Again, we report Cohen’s *d* as a measure of effect size for the main variables of interest.

#### Step 3 (Before the TP Offer)

In step 3 the results show no main effect for process frame with regard to the concessions on price ($F = 0.44; p > 0.1$), license contract duration (LCD) ($F = 0.17; p > 0.1$), online storage (OS) ($F = 0.01; p> 0.1$), and the sum of concessions ($F = 0.25; p > 0.1$). We also find no main effect for salience with regard to the concessions on price ($F = 0.44; p > 0.1$), LCD ($F = 0.05; p > 0.1$), OS ($F = 0.01; p > 0.1$), and the sum of concessions ($F = 0.34; p> 0.1$). Thus, in step 3 we find no difference with regard to concession behavior for the two variables (see Fig. 4d–f). Similarly, the payoffs do not differ for price (process frame: $F = 0.02; p > 0.1$; salience: $F = 1.16; p > 0.1$) and LCD (process frame: $F = 0.41; p> 0.1$; salience: $F = 1.83; p > 0.1$). However, we do find a significant effect for the process frame at the 10%-level for OS (process frame: $F = 3.27; p < 0.1$; salience: $F = 0.01; p > 0.1$).

#### Steps 4 and 5 (Response to Turning Point Offer)

As described in the method section, the alleged counterpart’s turning point offer consisted of a major concession on price (a high priority issue for the participant) and value claiming on LCD (a low priority issue for the participant). Thus, the turning point offer resembles a logrolling offer with the potential to turn the negotiation around. If negotiators recognize and embrace the turning point offer, they should change the structure of their counteroffer accordingly. The results show no significant effects of the treatments on overall payoffs (process frame: $F = 0.01; p> 0.1$; salience: $F = 1.89; p > 0.1$) and overall concessions (process frame: $F = 1.03; p > 0.1$; salience: $F = 0.01; p> 0.92$) as responses to the turning point offer. Also the results for the subsequent interaction step 5 reveal no significant difference with regard to the overall payoffs (process frame: $F = 0.05; p > 0.1$; salience: $F = 2.28; p > 0.1$) and concessions (process frame: $F = 0.46; p > 0.1$; salience: $F = 0.01; p > 0.1$) for both variables.

We do however find effects for the process frame and salience of the turning point offer for both the concessions on LCD (process frame: $F = 15.49; p < 0.01$; Cohen’s *d* = 0.74; salience: $F = 4.33; p < 0.05$; Cohen’s $d = 0.36$) as well as the offered payoffs (process frame: $F = 8.10; p< 0.01$; Cohen’s $d = 0.43$; salience: $F = 5.35; p< 0.05$; Cohen’s $d = 0.42$)^2^. Furthermore, the payoff differences persist to the interaction step following the response to the turning point offer as shown by the significant effects of process frame ($F = 26.14; p < 0.01$; Cohen’s $d = 0.96$) and salience ($F = 5.90; p < 0.05$; Cohen’s $d = 0.40$). The results reveal no significant interaction effect for LCD, neither for the payoffs ($F = 0.24; p > 0.1$) nor for the concession ($F= 0.03; p > 0.1$). Finally, results show that the process frame has a significant effect on the concession on price at the 10%-level in response to the turning point offer (process frame: $F = 3.38; p < 0.1$; Cohen’s $d = 0.42$; salience: $F = 0.56; p > 0.1$; Cohen’s $d = 0.20$) as well as on the payoffs in the subsequent interaction step (process frame: $F = 3.52; p < 0.1$; Cohen’s *d* = 0.21; salience: $F = 0.98; p > 0.1$; Cohen’s $d = 0.20$).

## Discussion

The results confirm the individual level hypotheses *H*1*a* and *H*2*a*. Both negotiating within an integrative-cooperative process frame and making the turning point offer more salient with a respective message increases the likelihood that negotiators interpret the departure as an opportunity to turn the negotiations around. Furthermore, it also increases the likelihood that negotiators respond in kind by matching the integrative pattern of the turning point offer confirming the interaction level hypotheses *H*1*b* and *H*2*b*. Notably, negotiators that embraced the turning point did not change the overall payoffs. They did not concede and lose value for themselves but adapted to the counterpart’s turning point offer by increasing their concessions on the issue the alleged counterpart signaled as being high priority and compensating with a large concession on the low-priority issue of price. Thus, our results show that the process frame and salience of the departure influence individual level as well as interactive level dynamics when negotiators enact the turning point.

We also proposed that salience would have a stronger effect within an integrative-cooperative context frame as individuals should be more receptive to a message highlighting mutual benefits (*H*3). However, the results did not show an interaction between salience and the process frame. The effect sizes also indicate that the process frame has a stronger effect than salience on negotiators’ interpretation of the turning point and on their behavior. A possible explanation is that the process frames mitigate salience: In the integrative-cooperative frame negotiators are already sufficiently motivated to detect potential areas of agreement and react to beneficial process changes. Thus, making the departure explicit provides only limited additional informational benefits for the negotiators. Conversely, in the distributive-competitive frame the beneficial turning point offer is evaluated in light of potential selfish motivations and strategic behavior. Thus, negotiators may be hesitant to fully embrace the departure, even when it is made explicit.

The findings have implications for turning point as well as (mis)matching research and provide further support for ROT. It adds to our knowledge about departures by highlighting the important roles played by attention and the social context: the same offer can result in different reactions from the counterpart depending on the process frame established and the salience of the offer. Turning points may be missed in a flurry of activity surrounding the negotiation. Attending to the departure is critical (Druckman 2004). But, attention is insufficient to produce a matching response. It needs to be accompanied by a cooperative frame leading to an interpretation of the offer as a genuine attempt to turn the negotiation around. A key to this finding is the trade-off potential in the offers. Our research has highlighted this element of integrative bargaining. Further research should explore other types of integrative agreements in a variety of negotiating environments.

Our results also contribute to the literature on (mis)matching by emphasizing the crucial role of social context in influencing negotiators’ concession behavior. Earlier research focused primarily on negotiators’ reactions to demands and concessions showing that they tend to exploit soft opponents that claim little (e.g., Bateman 1980; Druckman and Bonoma 1976; Yukl 1974b). This mismatching behavior, however, is attenuated by information about the counterpart’s limits and information about the outcomes that the counterpart achieves from the available alternatives (Liebert et al. 1968; Pruitt and Syna 1985; Yukl 1974a). Knowledge about the counterpart’s limits and preferences allows the negotiator to identify equal outcomes for both parties and decreases the likelihood of mismatching (Pruitt and Syna 1985). In absence of this information, negotiators have to rely on other cues for their counteroffers (Pietroni et al. 2008; Van Kleef et al. 2004a).

The findings extend previous research on (mis)matching behavior by identifying the process frame and salience as additional critical source of information affecting negotiators’ choice to match or mismatch. Negotiators may not be willing to exchange full information about their limits and preferences increasing the likelihood for mismatching. Other factors may compensate for this lack of disclosure. We have shown that salience and an integrative process frame promotes matching without full information. This is particularly important in the context of turning points. Synchronized responding to the departure can propel negotiators towards an agreement (Druckman 2004; Griessmair et al. 2015) while mismatching a conciliatory turning point offer may forfeit this opportunity.

More generally, our results provide evidence of the role of integrative-cooperative communication on the effectiveness of soft- as opposed to hardline bargaining. Results of meta-analyses conducted by Allen et al. support aspiration theory’s prediction of mismatching behavior and “the hardline bargaining strategy as the most effective for maximizing individual payoffs” (1990: 100). Yet, the authors stress that the superiority of this strategy may be a result of the experimental conditions, namely, distributive settings with no possibility to communicate, rather than the ineffectiveness of softball strategies and matching per se. They argue that “(s)oftline bargaining designed to induce a reciprocity norm needs open communication to persuade the other that the bargainer is conceding ... from a desire for more cooperative interaction” (Allen et al. 1990: 101). Both the integrative-cooperative process frame and salience communicated the intention to cooperate and, as a result, negotiators tended to reciprocate rather than exploiting the cooperative offer as suggested by aspiration theory. Thus, our findings provide initial evidence for the proposition that, when paired with appropriate communication, soft-line strategies induce reciprocity and may be more effective than hardline strategies.

This is in line with relational order theory (ROT) (Donohue 1998; Donohue and Hoobler 2002; Donohue and Roberto 1993). Both ROT and our findings suggest that it may be necessary to establish the appropriate frame before engaging in the talks. Donohue and Roberto (1993) conclude that police negotiators in hostage negotiations need to build a conducive relational context in which affiliation and interdependence are promoted before substantive proposals are made; a premature agreement may place the hostages at risk. Similarly, our findings show that introducing a turning point offer is more likely to be embraced by the counterpart and direct the negotiation toward a mutually beneficial agreement when the appropriate process frame has been established. Taken together, the present and previous studies support the idea that the effectiveness of specific negotiation behavior—proposing resolutions in hostage negotiations, introducing a turning point offer, or employing soft line strategies—is contingent on the communicative context or frame that has been established beforehand.

The findings also have practical implications for negotiators. Previous research has shown the benefits of employing integrative bargaining strategies (Olekalns et al. 2003; Olekalns and Weingart 2003; Weingart and Olekalns 2004; Weingart et al. 2004) and increasing the clarity of communication (Liu et al. 2010). The present study further confirms the value of these strategies for negotiators and extends it to the context of process frames and turning points. The process frame can be actively shaped and influenced by the negotiators. By creating a positive process frame, negotiators can lay the groundwork for successful departures that ultimately increase the odds for reaching a mutually beneficial agreement. An integrative-cooperative process frame may have additional benefits not considered in our experimental study. For instance, an integral part of integrative strategies is asking for and giving information about priorities and issue preferences (Weingart et al. 2004). Thus, employing integrative strategies should increase knowledge about the counterpart’s utility structure and promote the awareness required for identifying and reacting to process changes and departures. Similarly, an integrative-cooperative frame may increase mutual trust between the parties, which has proven to be an important facilitator for successfully enacting departures (Druckman et al. 2009).

Finally, many turning points studies have been conducted using retrospective case studies (for notable exceptions, see, Druckman and Olekalns 2013a; Druckman et al. 2009). This bolsters external validity as analyses are performed on real, often high level negotiations. As noted by Druckman and Olekalns, however, the “lack of control over events renders the analysis limited in terms of providing explanations for the occurrence of turning points” (2013b: 336). Our experimental design allowed us to control for the type, timing, and context of the departure as well as keeping the turning point offer constant in all treatments, thereby strengthening the arguments for causality However, the simulated counterpart comes at the cost of the external validity gained with the analyses of cases as it prevents a natural interaction to unfold. Since the relative strengths and weaknesses of the two approaches are complementary, Druckman and Olekalns (2013b) suggest that experimental studies of turning points should be complemented by retrospective case studies and vice versa. Thus, investigating the role of awareness, salience, and process frames in field settings may be a fruitful avenue for future research.

## Footnotes

1 The messages have been tested in a previous study. For more information on the content and design of the messages, see Griessmair and Seferagic (2014).

2 We also performed regressions controlling for the offered payoffs on price, LCD, and OS in step 3. Results still show significant effects for the process frame (payoff LCD: $\beta = -8.03$, SE = 2.16, $p < 0.01$; concession LCD: $\beta = 9.65$, SE = 2.21, $p < 0.01$) and message (payoff LCD: $\beta = -3.93$, SE = 2.18, $p < 0.1$; concession LCD: $\beta = 5.68$, SE = 2.21, $p< 0.05$).
